# PTEN-induced kinase 1 gene single-nucleotide variants as biomarkers in adjuvant chemotherapy for colorectal cancer: a retrospective study

**DOI:** 10.1186/s12876-023-02975-1

**Published:** 2023-10-02

**Authors:** Yoshiaki Mihara, Masataka Hirasaki, Yosuke Horita, Takashi Fujino, Hisayo Fukushima, Yasuo Kamakura, Kousuke Uranishi, Yasumitsu Hirano, Shomei Ryozawa, Masanori Yasuda, Yoshinori Makino, Satomi Shibazaki, Tetsuya Hamaguchi

**Affiliations:** 1 Department of Medical Oncology, Gastroenterological Oncology, Saitama Medical University International Medical Center, 1397-1 Yamane, Hidaka, Saitama 350-1298 Japan; 2 Department of Clinical Cancer Genomics, Saitama Medical University International Medical Center, 1397-1 Yamane, Hidaka, Saitama 350-1298 Japan; 3 Division of Biomedical Sciences, Research Center for Genomic Medicine, Saitama Medical University, 1397-1 Yamane, Hidaka, Saitama 350-1298 Japan; 4 Department of Gastroenterological Surgery, Lower Gastrointestinal Tract Surgery, Saitama Medical University International Medical Center, 1397-1 Yamane, Hidaka, Saitama 350-1298 Japan; 5 Department of Gastroenterology, Saitama Medical University International Medical Center, 1397-1 Yamane, Hidaka, Saitama 350-1298 Japan; 6 Department of Diagnostic Pathology, Saitama Medical University International Medical Center, 1397-1 Yamane, Hidaka, Saitama 350-1298 Japan; 7 Community Health Science Center, Saitama Medical University, 29 Morohongou, Iruma District, Moroyama Town, Saitama 350-0495 Japan

**Keywords:** Colorectal cancer, 5-FU, Adjuvant chemotherapy, Biomarker, PINK1

## Abstract

**Background:**

Fluoropyrimidine-based postoperative adjuvant chemotherapy is globally recommended for high-risk stage II and stage III colon cancer. However, adjuvant chemotherapy is often associated with severe adverse events and is not highly effective in preventing recurrence. Therefore, discovery of novel molecular biomarkers of postoperative adjuvant chemotherapy to identify patients at increased risk of recurrent colorectal cancer is warranted. Autophagy (including mitophagy) is activated under chemotherapy-induced stress and contributes to chemotherapy resistance. Expression of autophagy-related genes and their single-nucleotide polymorphisms are reported to be effective predictors of chemotherapy response in some cancers. Our goal was to evaluate the relationship between single-nucleotide variants of autophagy-related genes and recurrence rates in order to identify novel biomarkers that predict the effect of adjuvant chemotherapy in colorectal cancer.

**Methods:**

We analyzed surgical or biopsy specimens from 84 patients who underwent radical surgery followed by fluoropyrimidine-based adjuvant chemotherapy at Saitama Medical University International Medical Center between January and December 2016. Using targeted enrichment sequencing, we identified single-nucleotide variants and insertions/deletions in 50 genes, including autophagy-related genes, and examined their association with colorectal cancer recurrence rates.

**Results:**

We detected 560 single-nucleotide variants and insertions/deletions in the target region. The results of Fisher’s exact test indicated that the recurrence rate of colorectal cancer after adjuvant chemotherapy was significantly lower in patients with the single-nucleotide variants (c.1018G > A [*p* < 0.005] or c.1562A > C [*p* < 0.01]) of the mitophagy-related gene PTEN-induced kinase 1.

**Conclusions:**

The two single-nucleotide variants of *PINK1* gene may be biomarkers of non-recurrence in colorectal cancer patients who received postoperative adjuvant chemotherapy.

**Supplementary Information:**

The online version contains supplementary material available at 10.1186/s12876-023-02975-1.

## Background

Colorectal cancer (CRC) is the second leading cause of cancer-related death worldwide [1]. Because the recurrence rate of stage III and high-risk stage II CRC is more than 30%, a fluoropyrimidine (5-FU)-based regimen is recommended as postoperative adjuvant chemotherapy [2, 3]. However, defining high-risk stage II CRC is challenging because the criteria vary between different societies, such as the American Society of Clinical Oncology, European Society for Medical Oncology, and National Comprehensive Cancer Network [4–6]. Circulating tumor DNA has been postulated as a prognostic factor for postoperative recurrence in stage II colon cancer but has not been considered for practical use because of the high costs and insufficient evidence supporting this postulation [7, 8]. Furthermore, the effectiveness of current adjuvant chemotherapy is unsatisfactory. 5-FU-based adjuvant chemotherapy without oxaliplatin reduces the recurrence rate by only 10% compared with surgery alone, with a relative risk reduction of approximately 17%–32% [2, 9]. Even with the addition of oxaliplatin to adjuvant chemotherapy, the recurrence rate is reduced by only 5% compared with surgery alone [3]. Administration of 5-FU-based chemotherapy is also problematic as it has caused severe toxicity in up to 30% of all patients [10, 11]. Therefore, the decision to use adjuvant chemotherapy in CRC is left to the attending physician [12]. Given the above information, finding a recurrence-prevention biomarker in patients receiving adjuvant chemotherapy for CRC is necessary to expedite and guide treatment decisions.

A systematic review of nine randomized controlled phase III trials has revealed that *KRAS* and *BRAF* mutations are possible predictors of poor prognosis for stage II/III colon cancer treated with adjuvant chemotherapy. However, *KRAS* mutations significantly decreased disease-free survival, whereas *BRAF* mutation did not decrease disease-free survival. In addition, the trials did not include rectal cancer [13]; therefore, our goal is to find a novel recurrence biomarker in patients receiving adjuvant chemotherapy for CRC.

Autophagy is a highly regulated process that degrades and recycles cellular components. The most important features of autophagy include the breakdown of proteins and organelles in the cell and recycling them as a new source of nutrition [14]. In human colon cancer cell lines, autophagy is activated by 5-FU treatment, and inhibition of autophagy significantly increases 5-FU-induced apoptosis. Therefore, autophagy is activated as a protective mechanism against 5-FU-induced apoptosis [15]. Mitophagy is a form of autophagy that allows mitochondria to maintain homeostasis and plays a role in the late stages of tumorigenesis by increasing cell resistance and promoting carcinogenesis. This process mediates chemotherapy resistance in various types of cancer [16]. The silencing of the BCL2/adenovirus E1B 19-kDa protein-interacting protein 3 (*BNIP3*) in CRC and the high expression of PTEN-induced putative kinase 1 (*PINK1*) in esophageal cancer are associated with resistance to 5-FU-based chemotherapy [17, 18]. Both *BNIP3* and *PINK1* are mitophagy-related genes. Therefore, we planned to establish a system that predicts the efficacy of postoperative 5-FU-based adjuvant chemotherapy in CRC using the single-nucleotide variants (SNVs) of autophagy- and mitophagy-related genes.

The aim of this study was to find new recurrence-prevention biomarkers by analyzing autophagy- and cancer-related genes in specimens from patients undergoing 5-FU-based adjuvant chemotherapy for CRC and examine the association between the results and recurrence.

## Materials and methods

### Tissue samples

A total of 84 analytic samples from surgical or biopsy specimens were collected from 84 patients who underwent radical surgery for CRC at Saitama Medical University International Medical Center between January and December 2016. One case was excluded because the specimen was too small; therefore, we used a metastatic lymph node instead of the primary tumor. In three cases, double carcinoma was observed. In such situations, the case with the largest tumor invasion depth was selected or if the depths were the same, the one with a lower differentiation was selected. We used hematoxylin–eosin-stained slides to identify the location of the tumor cells in the tissue specimen both visually and microscopically by consulting the pathologist.

All patients underwent curative surgery followed by postoperative 5-FU-based adjuvant chemotherapy. Postoperative adjuvant chemotherapy consisted of regimens based on 5-FU: S-1; capecitabine; tegafur–uracil plus leucovorin calcium; oxaliplatin combinations such as FOLFOX (5-FU, levofolinate, and oxaliplatin), CAPOX (capecitabine and oxaliplatin), and SOX (S-1 and oxaliplatin); and oral uracil and tegafur plus leucovorin. Recurrence was defined as the date when CRC recurrence was confirmed via imaging (computed tomography, magnetic resonance imaging, and positron emission tomography), endoscopy, or clinical examination. The follow-up period for monitoring recurrence was within 5 years after surgery. Clinical information was obtained by reviewing medical records and pathology reports (Table 1).

**Table 1:** Clinical characteristics of the patients included in this study

	Recurrence (*n* = 27)	Non-recurrence (*n* = 57)	*p*-value
**Sex**			0.64
Male (%)	16 (59.3)	30 (52.6)	
Female (%)	11 (40.7)	27 (47.4)	
**Age (year)**			0.95
Median (range)	65 (38–79)	67 (40–80)	
**Pathological histotype**			0.48
Non poor (%)	18 (66.7)	33 (57.9)	
Poor (%)	9 (33.3)	24 (42.1)	
**Location**			0.62
Right (%)	7 (25.9)	19 (33.3)	
Left (%)	20 (74.1)	38 (66.7)	
**Depth**			0.23
< T4 (%)	20 (74.1)	49 (86.0)	
≥ T4 (%)	7 (25.9)	8 (14.0)	
**Stage**			0.14
II (%)	5 (18.5)	4 (7.0)	
III (%)	22 (81.5)	53 (93.0)	
**Smoking status**			0.64
Brinkman index			
≥ 400 (%)	12 (44.4)	22 (64.7)	
< 400 (%)	15 (55.6)	35 (70.0)	
**Alcoholic drinking**			0.66
Habitual drinker^a^ (%)	1 (3.7)	5 (8.8)	
Non-habitual drinker (%)	26 (96.3)	52 (91.2)	
**Adjuvant regimen**			0.72
Oxaliplatin combination^b^ (%)	8 (38.1)	13 (61.9)	
Cape (%)	7 (24.1)	22 (75.9)	
S-1 (%)	7 (35.0)	13 (65.0)	
UFT + LV (%)	5 (35.7)	9 (64.3)	

In addition to sex, age, and life history, the high-risk factors for recurrence and regimens for Stage II colorectal cancer are also indicated. Fisher’s exact test was used to compare the recurrence and non-recurrence groups

*Cape* capecitabine, *UFT* + *LV* oral uracil and tegafur (UFT) plus leucovorin (LV)

^a^Habitual drinker: drinking > 60 g of ethanol per day

^b^Oxaliplatin combination: FOLFOX (5-FU, levofolinate, oxaliplatin):1, CAPOX (capecitabine, oxaliplatin):18, SOX (S-1, oxaliplatin):1

### Statistical analysis

Fisher’s exact test was performed to determine significant associations between gene SNVs and cancer recurrence and non-recurrence (R package; https://bioconductor.org/packages/release/-bioc/html/edgeR.html). Logistic regression was used to validate confounding factors, Kaplan–Meier method was used to analyze the overall survival, and Student’s t-test and Wilcoxon rank sum test were conducted to determine the means of the two groups using the JMP Pro 16 software (SAS Institute Inc., Cary, NC, USA). All statistical tests were two-sided, and *p* < 0.05 was considered significant.

### DNA extraction, quantification, and quality control

Samples from 84 patients were analyzed. The cancerous areas were assessed and recovered using previously reported methods [19]. Chromosomal DNA was isolated from formalin-fixed paraffin-embedded (FFPE) colorectal adenocarcinoma samples using the QIAamp DNA FFPE Tissue Kit (QIAGEN, Hilden, Germany) according to the manufacturer’s instructions. DNA concentration was determined by measuring the fluorescence using the Qubit dsDNA HS kit (Thermo Fisher Scientific, Waltham, MA, USA).

### Target sequencing in our clinical CRC cases

We selected 50 autophagy-related genes and CRC-associated genes and identified the SNVs and insertion/deletions (INDELs) using targeted enrichment sequencing (see Additional file 1: Table S1). Several genes are required for the formation of autophagosomes. They can be broadly classified into the following functional groups: three genes (*PIK3R4*, *BECN1*, and *ATG14*) that contribute to “Vps34 PI3 kinase complex” formation, seven genes (*MAP1LC3A*, *ATG3*, *ATG4A*, *ATG4B*, *ATG4C*, *ATG4D*, and *ATG7*) that contribute to the “Atg8-conjugation system,” five genes (*ATG5*, *ATG10*, *ATG12*, *ATG16L1*, and *ATG16L2*) involved in the “Atg12-conjugation system,” eight genes (*ULK1*, *ATG13*, *RB1CC1*, *MTOR*, *RPTOR*, *DEPTOR*, *AKT1S1*, and *PTEN*) that are needed for the formation of the “Atg1 protein kinase complex,” and two genes (*ATG9A* and *ATG9B*) that are important for the “Atg9 and Atg2-Atg18 complex” [20]. In addition, mitophagy is a selective mechanism responsible for mitochondrial degradation induced via autophagy and is involved in the metabolism of old mitochondria. Eight genes (*PINK1*, *PRKN*, *BNIP3*, *BNIP3L*, *FUNDC1*, *OPTN*, *BCL2L13*, and *CALCOCO2*) contribute to the “mitophagy receptor” [20]. It was reported that *KRAS*-induced autophagy proceeds via the upregulation of the MEK/ERK pathway in colon models and that *KRAS* and autophagy contribute to CRC cell survival during starvation. Ten genes (*KRAS*, *NRAS*, *HRAS*, *ARAF*, *BRAF*, *RAF1*, *MAP2K1*, *MAP2K2*, *MAPK1*, and *MAPK3*) contribute to the “RAS-MEK/ERK pathway” [21]. Six genes (*APC*, *CTNNB1*, *ERBB2*, *SMAD4*, *PIK3CA*, and *TP53*) recognized to be mutated in CRC were selected as CRC-related genes [22]. Target regions were designed to enrich the exonic regions and exon–intron junctions of all 50 genes (see Additional file 1: Table S1). The mean percentile of covered target regions was 98.49%.

### Targeted capture and sequencing

A library of the entire genomic sequence of all 50 known genes (see Additional file 1: Table S1) was prepared using HaloPlex Target Enrichment kits (Agilent Technologies, Santa Clara, CA, USA) according to the manufacturer’s instructions. For each library, the confirmation of enrichment and the brief quantification of the enriched target DNA were performed using the High Sensitivity D1000 Screen Tape System (Agilent Technologies). The pooled samples with different indices for multiplex sequencing were measured using the library quantification kit (Kapa Biosystems, Wilmington, MA, USA) to obtain molar concentrations. High-throughput sequencing was performed with 150-bp paired-end reads on a MiSeq or NextSeq platform (Illumina, San Diego, CA, USA) for each pooled sample according to the manufacturers’ protocols.

### Data analysis for next generation sequencing

The raw sequence read data passed the quality checks in FastQC (http://www.bioinformatics.babraham.ac.uk/projects/fastqc). Read trimming via base quality was performed using FASTX-toolkit v.0.0.14 [23]. Read alignments to the UCSC hg38 reference genome were performed using the Burrows–Wheeler Aligner [24]. Non-mappable reads were removed using SAMtools [25]. After filtering these reads, the Genome Analysis Toolkit (GATK) was used for local realignment and base quality score recalibration. For detecting SNVs and small INDELs, we applied the GATK multiple-sample calling protocol [26]. The coverage of the targeted regions was estimated using the GATK DepthOfCoverage. In this experiment, we used SelectVariants to select variants with “DP > 10” (depth of coverage greater than 10 ×). The detected variants were annotated using ANNOVAR, and pathogenicity was assessed using the ClinVar_20210501 database [27].

### Sanger sequencing analysis

Sanger sequencing analysis was conducted to confirm the location of specific SNVs in the detected genes. PCR was performed using the PrimeSTAR Max DNA polymerase system (Takara Bio, Kusatsu, Japan). Thereafter, PCR-amplified products were extracted using the QIAquick Gel Extraction Kit (QIAGEN). Sequencing in the reverse direction was undertaken according to the manufacturer’s instructions (BigDye; Applied Biosystems, Warrington, UK). Sequencing of the products was performed using the ABI 3500 automated DNA sequencer (Applied Biosystems).

### Supplementary information

The targeted genes in the selected clinical colorectal cancer cases (see Additional file 1: Table S1), correlation between all SNVs and recurrence rate (see Additional file 1: Table S2) and correlation between ClinVar-based pathogenic SNVs and recurrence rate (see Additional file 1: Table S3) are described in Additional file 1.

## Results

### Clinical characteristics

The clinical characteristics of the 84 CRC patients receiving adjuvant chemotherapy are shown in Table 1. Stage II patients exhibited a higher recurrence rate than Stage III patients did; however, the difference was insignificant. There were no significant differences in other clinical characteristics between the recurrence and non-recurrence groups. Of the 84 patients, the total number of patients exhibiting recurrence was 27 (32.1%). Moreover, 22 of the 27 recurrent cases (81.5%) were confirmed through computed tomography, and the sites of recurrence were liver in 9 cases, lung in 6, peritoneum in 4, lymph node in 3, local in 2, and ovary, bone, and tumor plug each in 1 patient each. In addition, 68 of the 84 patients (81.0%) received 5-FU-based adjuvant chemotherapy for the standard 6 months, while 16 (19.0%) did not complete 6 months of adjuvant chemotherapy; 11 of these 16 patients discontinued adjuvant chemotherapy because Grade 3 or higher adverse events occurred, and the remaining patients discontinued because of personal or social reasons.

### Quality assessment

**Fig. 1:**
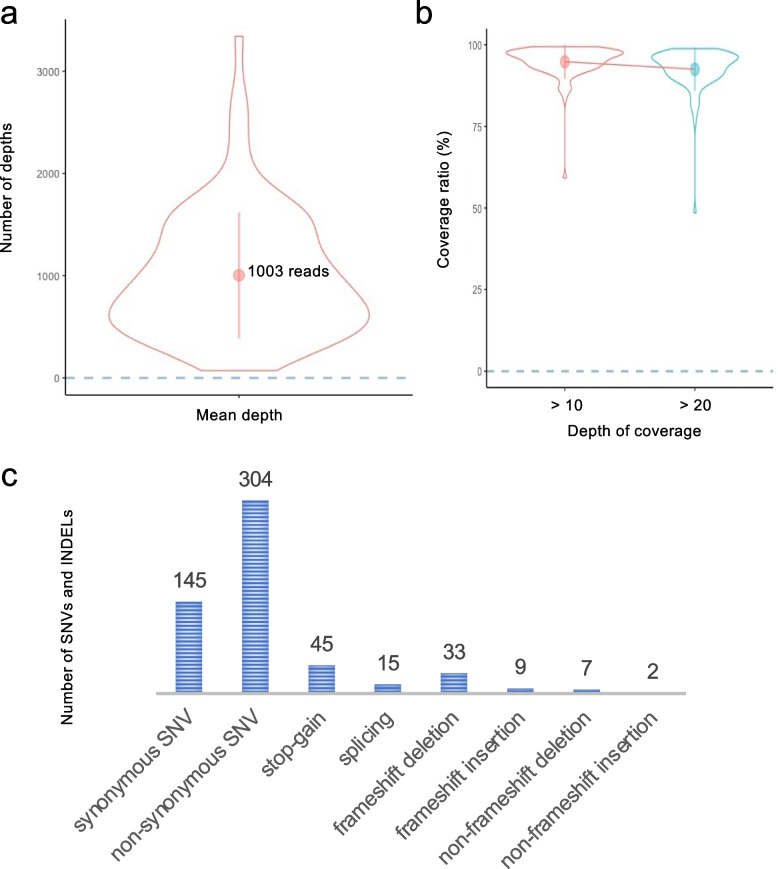
Results of the original target enrichment sequencing in our CRC clinical cases. **a** The violin plot depicts the distribution of the mean depth for each of the 84 multiplexed samples. **b** The violin plot depicts the distribution of the coverage ratio for each of the 84 multiplexed samples. Percentage of regions with a depth of coverage greater than 10 × for red and greater than 20 × for green. **c** The number of SNVs or INDELs identified by the original target enrichment sequencing is shown. Classification was performed by variant type. SNV: single-nucleotide variant, INDEL: insertion/deletion

A median of 3,493,323 sequence-mapped reads were obtained per sample (range: 437,058–6,034,492 reads/sample). Among the designed target bases, 93.2% (range: 58.3%–97.9% per sample) had at least 10-fold coverage, with a mean coverage of 1003-fold (range: 71- to 3342-fold) per nucleotide in the coding region of the target gene (Fig. 1a and b). Although one sample with a significantly low coverage was found, it was not expected to have a significant effect on the overall results because the GATK multiple-sample calling protocol was used to detect SNVs and small INDELs during the sequencing analysis. Furthermore, the SelectVariants option was applied to remove data with a depth of coverage less than 10.

### Breakdown of SNVs and INDELs

The original target enrichment sequencing for cases treated with postoperative adjuvant chemotherapy showed that 560 SNVs or INDELs were detected in the target region (Fig. 1c). There were 304 non-synonymous SNVs in the amino acid sequences: 33 had frameshift deletions and 9 had frameshift insertions (Fig. 1c). SNVs showing the stop-gain variant were found in 45 locations (Fig. 1c).

### Individual SNVs obtained by target enrichment sequencing

**Fig. 2:**
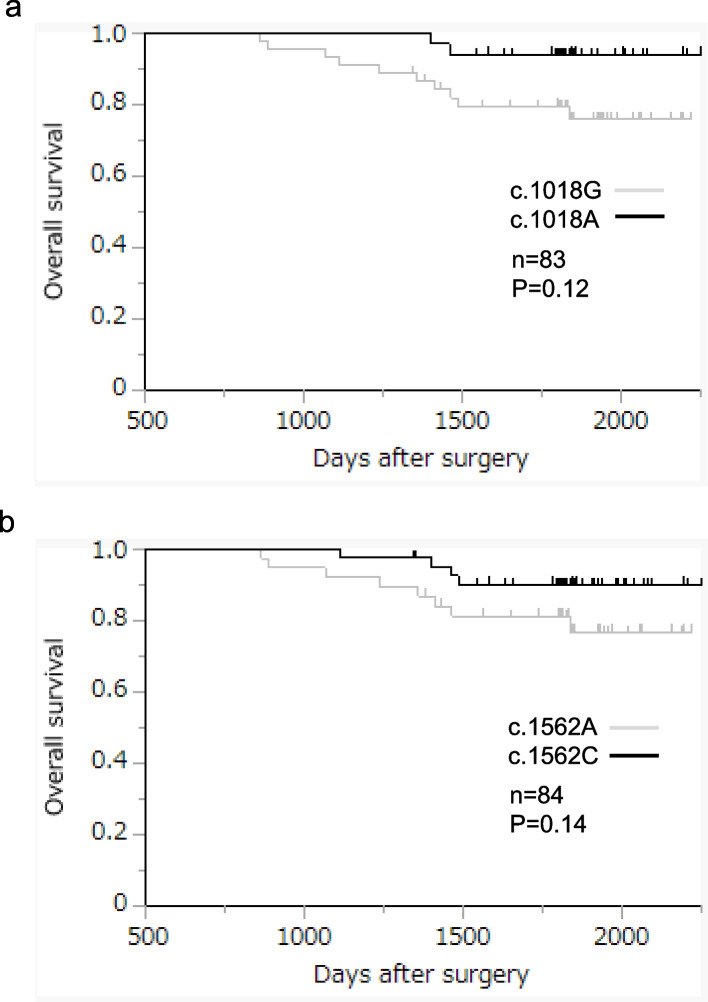
Relationship between SNVs of *PINK1* (c.1018G > A and c.1562A > C) and CRC prognosis with 5-FU-based adjuvant chemotherapy: overall survival with or without (**a**) c.1018G > A or (b) c.1562A > C. The analyzed specimens were 84 and 83 for (a) c.1018G > A and (**b**) c.1562A > C, respectively. In one case, the sample of (**b**) c.1018G > A was not analyzed because of an inappropriate specimen status. No statistically significant relationship was found between the two SNVs in *PINK1* and overall survival. CRC: colorectal cancer

The samples of 84 patients were included in the analysis. The results of Fisher’s exact test performed on the 560 SNVs or INDELs indicated that the variants were lower in the recurrence group (*n* = 27) than in the non-recurrence group (*n* = 57). A significant difference of less than *p* < 0.05 was found in two non-synonymous SNVs: *PINK1* c.1018G > A and *PINK1* c.1562A > C and three synonymous SNVs (*KRAS* c.519 T > C, *DEPTOR* c.135C > T, and *OPTN* c.102G > A) (Table 2). However, neither the c.1018G > A nor the c.1562A > C SNV in *PINK1* showed a statistically significant relationship with the overall survival (OS; Fig. 2a and b). Logistic regression was conducted to test for confounding effects on the relationship between the two *PINK1* SNVs (c.1018G > A [*p* = 0.01] and c.1562A > C [*p* = 0.01]) and the non-recurrence group, and showed no significant influence of pathological histotype, location, depth, stage, or adjuvant regimen on either SNV (Table 3 and 4). The presence or absence of any SNV was not correlated with the occurrence of Grade 3 or higher adverse events (c.1018G > A [*p* = 0.28] and c.1562A > C [*p* = 0.13]) or with the duration of adjuvant chemotherapy (c.1018G > A [*p* = 0.92] and c.1562A > C [*p* = 0.92]).

**Table 2:** Results of target enrichment sequencing

				Recurrence		Non-recurrence		
Gene symbol	Exonic function	Nucleotide change	Aa change	RefSeq (n)	AltSeq (n)	RefSeq (n)	AltSeq (n)	*p*-value
*PINK1*	non-synonymous SNV	NM_032409:c.1018G > A	p.A340T	21 (80.8)	5 (19.2)	27 (47.4)	30 (52.6)	0.0045
*PINK1*	non-synonymous SNV	NM_032409:c.1562A > C	p.N521T	19 (70.4)	8 (29.6)	22 (38.6)	35 (61.4)	0.0098
*KRAS*	synonymous SNV	NM_001369787:c.519 T > C	p.D173D	16 (59.3)	11 (40.7)	47 (82.5)	10 (17.5)	0.0311
*DEPTOR*	synonymous SNV	NM_022783:c.135C > T	p.H45H	24 (88.9)	3 (11.1)	57 (100)	0 (0)	0.0307
*OPTN*	synonymous SNV	NM_021980:c.102G > A	p.T34T	14 (51.9)	13 (48.1)	44 (77.2)	13 (22.8)	0.0245

Percentage in parentheses

Eighty-four patients were included in the analysis; Fisher’s exact test of 560 SNVs or INDELs showed 5 SNVs with *p* < 0.05

*Aa change* amino acid change, *RefSeq* allele in the reference genome, *AltSeq* Alt, any other allele found at that locus

**Table 3:** Confounding factors that influence the recurrence prevention effectiveness of the c.1018G > A SNV in* PINK1*

	Recurrence (*n* = 26^a^)	Non-recurrence (*n* = 57)	*p*-value
***PINK1***			
c.1018A (*n* = 35) (%)	5 (19.2)	30 (52.6)	**0.01**
c.1018G (*n* = 48) (%)	21 (80.8)	27 (47.4)	
**Pathological histotype**			0.89
Non poor (%)	17 (65.4)	33 (57.9)	
Poor (%)	9 (34.6)	24 (42.1)	
**Location**			0.53
Right (%)	7 (26.9)	19 (33.3)	
Left (%)	19 (73.1)	38 (66.7)	
**Depth**			0.79
< T4 (%)	19 (73.1)	49 (86.0)	
≥ T4 (%)	7 (26.9)	8 (14.0)	
**Stage**			0.23
II (%)	5 (19.2)	4 (7.0)	
III (%)	21 (80.8)	53 (93.0)	
**Adjuvant regimen**^**b**^			0.95
Oxaliplatin combination (%)	7 (26.9)	13 (22.8)	
Others (%)	19 (73.1)	44 (77.2)	

Logistic regression was performed to test for confounding effects on the relationship between a *PINK1* SNV (c.1018G > A) and non-recurrence of CRC

^a^Neither c.1018A nor c.1018G were detected in one case of recurrence

^b^Oxaliplatin combination: FOLFOX (5-FU, levofolinate, oxaliplatin):1, CAPOX (capecitabine, oxaliplatin):18, SOX (S-1, oxaliplatin):1

**Table 4:** Confounding factors that influence the recurrence prevention effectiveness of the c.1562A > C SNV in* PINK1*

	Recurrence (*n* = 27)	Non-recurrence (*n* = 57)	*p*-value
***PINK1***			
c.1562C (*n* = 43) (%)	8 (29.6)	35 (61.4)	**0.01**
c.1562A (*n* = 41) (%)	19 (70.4)	22 (38.6)	
**Pathological histotype**			0.99
Non poor (%)	18 (66.7)	33 (57.9)	
Poor (%)	9 (33.3)	24 (42.1)	
**Location**			0.42
Right (%)	7 (25.9)	19 (33.3)	
Left (%)	20 (74.1)	38 (66.7)	
**Depth**			0.55
< T4 (%)	20 (74.1)	49 (86.0)	
≥ T4 (%)	7 (25.9)	8 (14.0)	
**Stage**			0.4
II (%)	5 (18.5)	4 (7.0)	
III (%)	22 (81.5)	53 (93.0)	
**Adjuvant regimen**^**a**^			0.96
Oxaliplatin combination (%)	8 (29.6)	13 (22.8)	
Others (%)	19 (70.4)	44 (77.2)	

Logistic regression was performed to test for confounding effects on the relationship between a *PINK1* SNV (c.1562A > C) and non-recurrence of CRC

^a^Oxaliplatin combination: FOLFOX (5-FU, levofolinate, oxaliplatin):1, CAPOX (capecitabine, oxaliplatin):18, SOX (S-1, oxaliplatin):1

### SNVs of *PINK1* gene

**Fig. 3:**
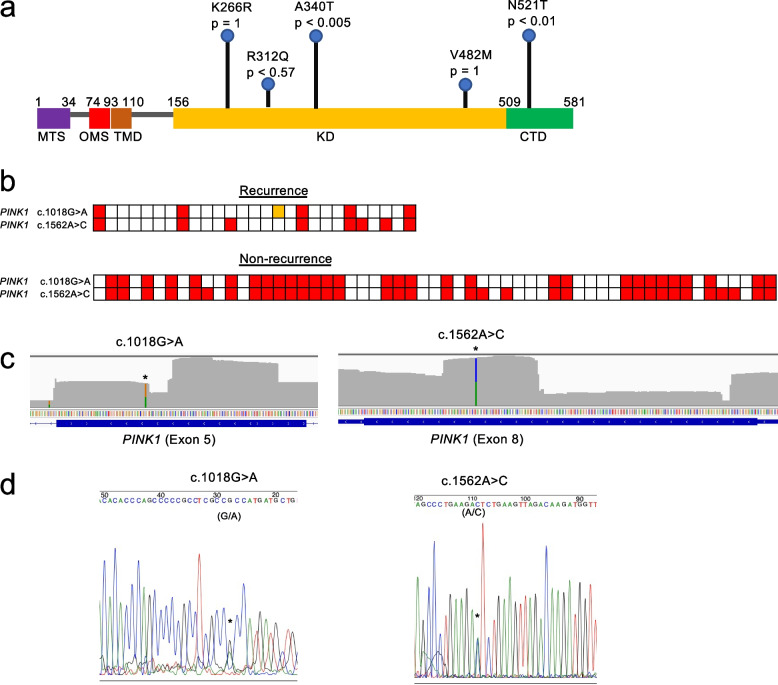
SNVs of *PINK1* gene detected in this study. **a** The full-length PINK1 can be divided into five structural and functional regions: MTS, OMS, TMD, KD, and CTD. p indicates the *p*-value. CTD: C-terminal domain, KD: kinase domain, MTS: mitochondrial targeting sequence, OMS: outer membrane localization signal, TMD: transmembrane domain. **b** Samples with AltSeq for *PINK1* are shown in red. Samples whose sequence reads did not satisfy the criteria are marked in yellow. **c**, **d** Sequencing chromatograms illustrating *PINK1* c.1018G > A and c.1562A > C. * indicates the location of the SNV. **c** Integrative Genomics Viewer screenshot of soft-clipped reads in exons 5 and 8 of *PINK1*. Green represents A, yellow represents G, and blue represents C. **d** Sanger sequencing data

The PINK1/Parkin pathway is the most studied pathway of mitophagy, and serine/threonine PINK1 is the initiator of this pathway [28]. In this target enrichment sequencing, four non-synonymous SNVs of PINK1 were found in the kinase domains (KDs) (Fig. 3a). One of the remaining SNVs was also found on the C-terminal domain sequence (Fig. 3a), which controls the structure of the KD and helps the kinase region identify the substrate [29]. In the current study, two SNVs (p.A340T and p.N521T) showed significant differences; however, we believe that significant differences might have been observed in other SNVs on the KD if the number of cases had been higher.

Two SNVs in the *PINK1* gene were found to be significantly identified in the non-recurrence group compared to the recurrence group. The SNVs were marked in red for each patient in which they were found, and c.1018G > A was always found in conjunction with c.1562A > C (Fig. 3b).

The results obtained by next generation sequencing of c.1562A > C and c.1018G > A of *PINK1* were visualized using the Integrative Genomics Viewer software (Fig. 3c). The same positions were reconfirmed by the Sanger sequencing method. The SNVs of *PINK1* (c.1018G > A and c.1562A > C) were amplified using specific primer sets. The primer set for c.1018G > A (annealing at 53 °C) included the forward primer (5′-TCGATGTGTGGTAGCCAGAG-3′) and reverse primer (5′-GATGCCCTGTTGAACCAGAT-3′). The primer set for c.1562A > C (annealing at 50 °C) included the forward primer (5′-CCGCAAATGTGCTTCATCTA-3′) and reverse primer (5′-AGCGTTTCACACTCCAGGTT-3′), and the overlap of G (black) and A (green) waveforms in c.1018 as well as that of A (green) and C (blue) waveforms in c.1562 (Fig. 3d) were observed.

### Correlation between pathogenic/likely pathogenic SNV and recurrence rate

Fisher’s exact test was performed in the recurrence and non-recurrence groups for each gene that showed non-synonymous SNVs resulting in amino acid substitutions or SNVs with significant effects such as frameshift deletion, frameshift insertion, stop-gain, and splicing. The results showed significant differences only for *PINK1* (see Additional file 1: Table S2, Fig. 3a).

Fisher’s exact test was performed for each gene that showed non-synonymous SNVs and was determined to be pathogenic/likely pathogenic in ClinVar, and no genes were significantly different in terms of the non-synonymous SNVs (see Additional file 1: Table S3).

## Discussion

The c.1018G > A and c.1562A > C of the mitophagy-related gene *PINK1* may be used as biomarkers of non-recurrence in CRC patients receiving postoperative adjuvant chemotherapy. Although there is a worldwide consensus on postoperative adjuvant chemotherapy for stage III and high-risk stage II CRC, several problems still exist. First, the criteria that define high-risk stage II CRC vary among academic societies. Second, adjuvant chemotherapy with 5-FU has limited efficacy in preventing recurrence [2, 8]. Third, 5-FU-based adjuvant chemotherapy causes severe toxicity [9, 10]. Although the current study does not provide a direct solution to these problems, the development of biomarkers for predicting the efficacy of adjuvant chemotherapy would prevent unnecessary administration, improve patients’ quality of life, and reduce costs; thus, this study offers indirect solutions. In our search for biomarkers, we focused on mitophagy-related genes because mitophagy has recently been associated with chemotherapy resistance.

Mitophagy is a unique autophagic action that removes damaged mitochondria and plays an important role in maintaining mitochondrial quality. The mitophagy pathways can be broadly classified into the PINK1-Parkin-mediated ubiquitin pathway and the FUNDC1/BNIP3/NIX receptor–receptor-mediated pathway [16]. Mitophagy inhibits early tumorigenesis and thus protects the normal cells. However, as cancer progresses, various genetic changes occur, resulting in the accumulation of impaired mitochondria, suppression of mitophagy, and promotion of tumorigenesis. Moreover, in advanced cancers, the rapid removal of mitochondria that has been damaged by the stress of chemotherapy via mitophagy is thought to promote cancer cell survival and result in drug resistance [17, 30].

Zhang et al. examined 451 patients with unresectable colon cancer treated with FOLFIRI (5-FU, levofolinate, and irinotecan) plus bevacizumab in two phase III trials and demonstrated that some single-nucleotide polymorphisms in *BNIP3* were predictors of satisfactory response to the regimen [31]. Another study analyzed 81 patients with unresectable CRC treated with 5-FU-based regimens and demonstrated that the loss of BNIP3 expression in cancer cells increased resistance to 5-FU-based drugs and worsened prognosis. These results suggested that BNIP3-related factors might predict drug resistance; however, in the current study, the SNVs of *BNIP3* were not correlated with recurrence rate. Although stage II colon cancer with high microsatellite instability may have a good prognosis, 5-FU adjuvant therapy is not effective in treating this cancer [32]. Therefore, favorable prognostic factors may not necessarily be effective in preventing recurrence, and the difference between unresectable cancer and postoperative recurrence may also influence the recurrence rate. In the current study, SNVs in *PINK1* were also associated with recurrence prevention but were not significantly associated with OS. No conclusions could be drawn owing to the small number of cases studied, and this study did not examine the relationship between SNVs and mutations. We believe that there was no correlation between SNVs of *KRAS* and *BRAF* and recurrence rates for the same reason.

In 159 patients with esophageal cancer who received 5-FU- and cisplatin-based preoperative chemotherapy, high expression of PINK1 was correlated with poor response to neoadjuvant chemotherapy, thus suggesting that PINK1-mediated mitophagy contributes to resistance to 5-FU-based neoadjuvant therapy [17].

We found a correlation between several SNVs of *PINK1* (c.1018G > A and c.1562A > C) and the non-recurrence rate. If we hypothesize that the SNVs of *PINK1* reduce mitophagic activity and result in low expression of PINK1, then the correlation between the SNVs of *PINK1* and lower recurrence rates is consistent with the finding that a high expression of *PINK1* in esophageal cancer during preoperative chemotherapy is correlated with poor efficacy. These results indicate that the SNVs of *PINK1* may reduce mitophagic activity, thus reducing chemotherapy resistance and enhancing the effect of 5-FU-based adjuvant. However, the biochemical significance of the two SNVs of *PINK1* has not been clarified.

This study is limited by (1) a small sample size, (2) an adjuvant chemotherapy regimen that is based on 5-FU but is not identical, (3) the lack of comparison with a group that received surgery alone, and (4) incomplete functional analysis of SNVs despite them being candidate biomarkers. However, the identified SNVs are more promising than other biomarkers because, unlike SNVs in *KRAS* and *BRAF*, they target the entire colon cancer population, including rectal cancer, and are not rare like *BRAF*, nor are they expensive like circulating tumor DNA. This is the first report of mitophagy-related SNVs as biomarkers for non-recurrence of CRC treated with postoperative adjuvant chemotherapy, and further research will deepen our knowledge on this topic.

## Conclusions

In summary, we attempted to identify new biomarkers for recurrence prevention by analyzing autophagy- and cancer-related genes in samples from patients who received 5-FU-based adjuvant chemotherapy for CRC. We found that the c.1018G > A and c.1562A > C SNVs of *PINK1* may be promising biomarkers for the favorable treatment effects of adjuvant chemotherapy in CRC. Further prospective studies are needed to understand their mechanisms of action.

### Supplementary Information

**Additional file 1: Table S1.** Targeted genes in the selected clinical colorectal cancer cases. Fifty selected autophagy-related and CRC-related genes are listed. The location and region of each gene and the coverage rate of the targeted sequencing for each region are shown. **Table S2.** Correlation between all SNVs and recurrence rate. Fisher’s exact test was performed for each gene that showed a similar SNV resulting in an amino acid substitution in the recurrence and non-recurrence groups. RefSeq.: allele in the reference genome, AltSeq.: Alt, any other allele found at that locus. **Table S3.** Correlation between ClinVar-based pathogenic SNVs and recurrence rates. Fisher’s exact test was performed for each gene that exhibited a non-synonymous SNV and was determined to be pathogenic/likely pathogenic by ClinVar. RefSeq: allele in the reference genome, AltSeq: Alt, any other allele found at that locus.

## Data Availability

Raw sequences were deposited in the NCBI Short Read Archive (SRA) database (http://www.ncbi.nlm.nih.gov/Traces/sra/) and the accession number was PRJNA1014496. However, the above correspondence table linking patient identification codes to personal information is not publicly available due to privacy and ethical constraints. When an application for secondary use of sequence data is submitted, we will ask the applicant to present the purpose of use, etc., and we will review the pros and cons and make a decision. The person who handles applications for use is Masataka Hirasaki (hirasaki@saitama-med.ac.jp).

## Abbreviations

5-FU: Fluoropyrimidine
BNIP3: BCL2/adenovirus E1B 19-kDa protein-interacting protein 3
CRC: Colorectal cancer
FFPE: Formalin-fixed paraffin-embedded
GATK: Genome Analysis Toolkit
KD: Kinase domain
OS: Overall survival
PINK1: PTEN-induced putative kinase 1
SNV: Single-nucleotide variant
