# Correction to: PTEN-induced kinase 1 gene single-nucleotide variants as biomarkers in adjuvant chemotherapy for colorectal cancer: a retrospective study

**DOI:** 10.1186/s12876-023-02994-y

**Published:** 2023-10-31

**Authors:** Yoshiaki Mihara, Masataka Hirasaki, Yosuke Horita, Takashi Fujino, Hisayo Fukushima, Yasuo Kamakura, Kousuke Uranishi, Yasumitsu Hirano, Shomei Ryozawa, Masanori Yasuda, Yoshinori Makino, Satomi Shibazaki, Tetsuya Hamaguchi

**Affiliations:** 1 Department of Medical Oncology, Gastroenterological Oncology, Saitama Medical University International Medical Center, 1397-1 Yamane, Hidaka, Saitama 350-1298, Japan; 2 Department of Clinical Cancer Genomics, Saitama Medical University International Medical Center, 1397-1 Yamane, Hidaka, Saitama 350-1298, Japan; 3 Division of Biomedical Sciences, Research Center for Genomic Medicine, Saitama Medical University, 1397-1 Yamane, Hidaka, Saitama 350-1298, Japan; 4 Department of Gastroenterological Surgery, Lower Gastrointestinal Tract Surgery, Saitama Medical University International Medical Center, 1397-1 Yamane, Hidaka, Saitama 350-1298, Japan; 5 Department of Gastroenterology, Saitama Medical University International Medical Center, 1397-1 Yamane, Hidaka, Saitama 350-1298, Japan; 6 Department of Diagnostic Pathology, Saitama Medical University International Medical Center, 1397-1 Yamane, Hidaka, Saitama 350-1298, Japan; 7 Community Health Science Center, Saitama Medical University, 29 Morohongou, Iruma District, Moroyama Town, Saitama 350-0495, Japan

**Correction to:** ***BMC Gastroenterol*** **23, 339 (2023).**

10.1186/s12876-023-02975-1.

Following publication of the original article [1], it was reported that there was a typographical mistake in the author list. Satomi Shibazaki’s name was erroneously given as Satomi Shizasaki.

The correct authorship is given in this Correction article and the original article [1] has been updated.
