# Correction: PTEN-induced kinase 1 gene single-nucleotide variants as biomarkers in adjuvant chemotherapy for colorectal cancer: a retrospective study

**DOI:** 10.1186/s12876-024-03154-6

**Published:** 2024-02-06

**Authors:** Yoshiaki Mihara, Masataka Hirasaki, Yosuke Horita, Takashi Fujino, Hisayo Fukushima, Yasuo Kamakura, Kousuke Uranishi, Yasumitsu Hirano, Shomei Ryozawa, Masanori Yasuda, Yoshinori Makino, Satomi Shibazaki, Tetsuya Hamaguchi

**Affiliations:** 1 Department of Medical Oncology, Gastroenterological Oncology, Saitama Medical University International Medical Center, 1397-1 Yamane, Hidaka, Saitama 350-1298 Japan; 2 Department of Clinical Cancer Genomics, Saitama Medical University International Medical Center, 1397-1 Yamane, Hidaka, Saitama 350-1298 Japan; 3 Division of Biomedical Sciences, Research Center for Genomic Medicine, Saitama Medical University, 1397-1 Yamane, Hidaka, Saitama 350-1298 Japan; 4 Department of Gastroenterological Surgery, Lower Gastrointestinal Tract Surgery, Saitama Medical University International Medical Center, 1397-1 Yamane, Hidaka, Saitama 350-1298 Japan; 5 Department of Gastroenterology, Saitama Medical University International Medical Center, 1397-1 Yamane, Hidaka, Saitama 350-1298 Japan; 6 Department of Diagnostic Pathology, Saitama Medical University International Medical Center, 1397-1 Yamane, Hidaka, Saitama 350-1298 Japan; 7 Community Health Science Center, Saitama Medical University, 29 Morohongou, Iruma District, Moroyama Town, Saitama 350-0495 Japan

**Correction: BMC Gastroenterol 23, 339 (2023)**

**https://doi.org/10.1186/s12876-023-02975-1**

Following publication of the original article [1] the authors reported an error in Fig. 2. A technical error (reversing the definition of "1" and "0" for censored cases and deaths) when creating the Kaplan–Meier survival curve resulted in an incorrect curve. Furthermore, the original Fig. 2 was missing a numerical value on the vertical axis. The figure legend was correct and is unchanged.

**Fig. 2:**
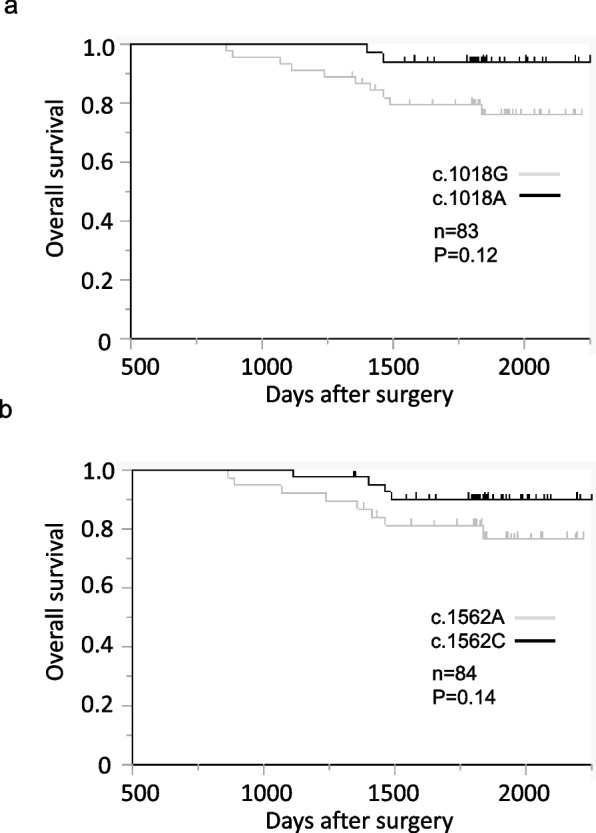
Relationship between SNVs of *PINK1* (c.1018G > A and c.1562A > C) and CRC prognosis with 5-FU-based adjuvant chemotherapy: overall survival with or without (**a**) c.1018G > A or (b) c.1562A > C. The analyzed specimens were 84 and 83 for (a) c.1018G > A and (**b**) c.1562A > C, respectively. In one case, the sample of (**b**) c.1018G > A was not analyzed because of an inappropriate specimen status. No statistically significant relationship was found between the two SNVs in *PINK1* and overall survival. CRC: colorectal cancer

The incorrect version of Fig. 2 was:
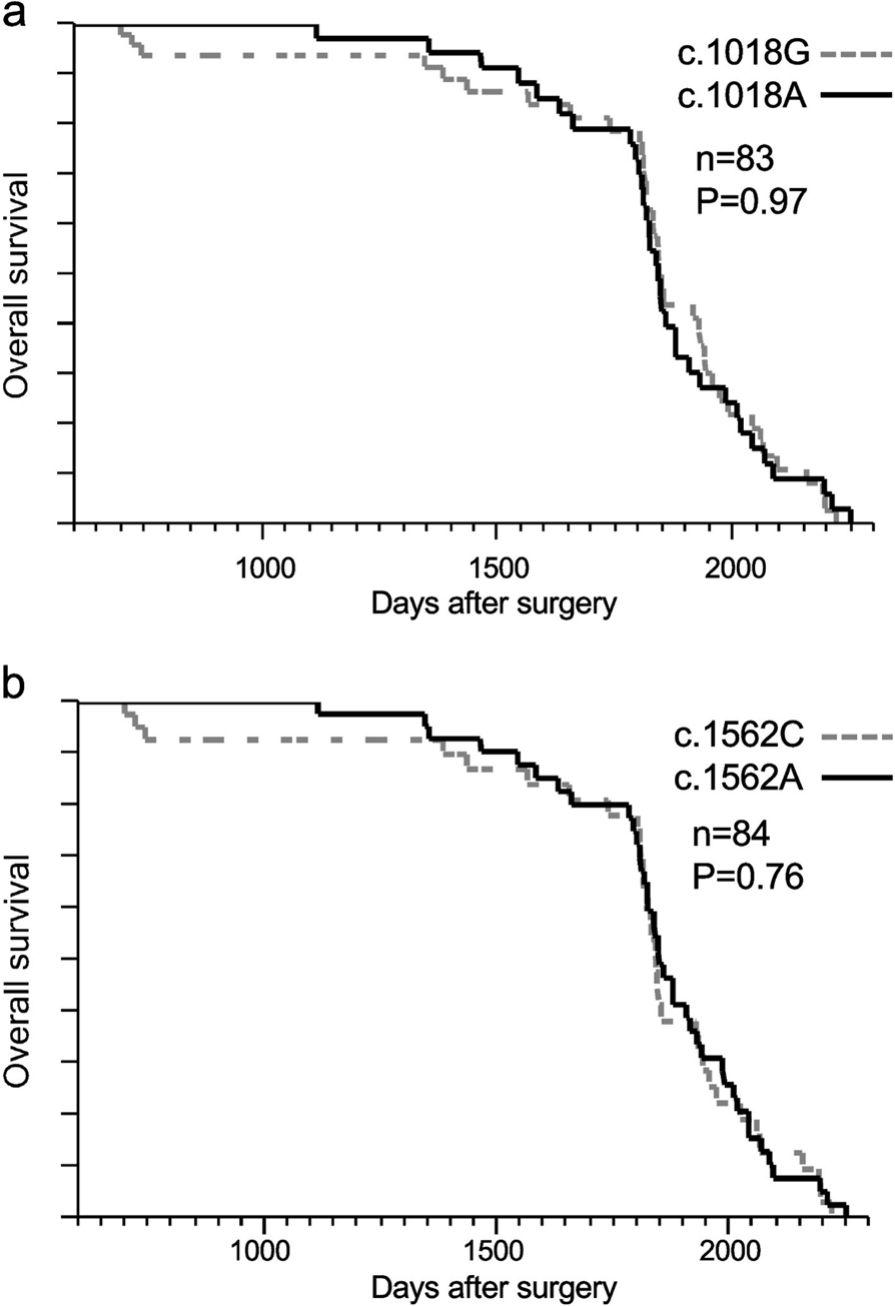


The correct version of Fig. 2 is:

The original article has been updated.
