# Supplementary material for: PTEN-induced kinase 1 gene single-nucleotide variants as biomarkers in adjuvant chemotherapy for colorectal cancer: a retrospective study
Source: BMC Gastroenterol. 2023 Oct 2;23:339. doi: 10.1186/s12876-023-02975-1 (PMC10544379; doi:10.1186/s12876-023-02975-1)
Supplement: Supplementary file 1 — Additional file 1: Table S1. Targeted genes in the selected clinical colorectal cancer cases. Fifty selected autophagy-related and CRC-related genes are listed. The location and region of each gene and the coverage rate of the targeted sequencing for each region are shown. Table S2. Correlation between all SNVs and recurrence rate. Fisher’s exact test was performed for each gene that showed a similar SNV resulting in an amino acid substitution in the recurrence and non-recurrence groups. RefSeq.: allele in the reference genome, AltSeq.: Alt, any other allele found at that locus. Table S3. Correlation between ClinVar-based pathogenic SNVs and recurrence rates. Fisher’s exact test was performed for each gene that exhibited a non-synonymous SNV and was determined to be pathogenic/likely pathogenic by ClinVar. RefSeq: allele in the reference genome, AltSeq: Alt, any other allele found at that locus. [file 12876_2023_2975_MOESM1_ESM.docx]

**PTEN-induced kinase 1 gene single-nucleotide variants as biomarkers in adjuvant chemotherapy for colorectal cancer**

**Journal Name:** BMC Gastroenterology

Yoshiaki Mihara, Masataka Hirasaki, Yosuke Horita, Takashi Fujino, Hisayo Fukushima, Yasuo Kamakura, Kousuke Uranishi, Yasumitsu Hirano, Shomei Ryozawa, Masanori Yasuda, Yoshinori Makino, Satomi Shibasaki, Tetsuya Hamaguchi

**Corresponding author:**

Masataka Hirasaki

Department of Clinical Cancer Genomics, Saitama Medical University International Medical Center, Hidaka, Saitama 350-1298, Japan

Email: hirasaki@saitama-med.ac.jp

Table S1**.** Targeted sequences in the selected clinical colorectal cancer cases

| **Symbol** | **Location (hg19)** | **Target region (bp)** | **Coverage region (%)** |
| --- | --- | --- | --- |
| RAS-MEK/ERK pathway | |  |  |
| *KRAS* | chr12:25362719–25398328 | 828 | 96.74 |
| *NRAS* | chr1:115251146–115258791 | 650 | 100 |
| *HRAS* | chr11:532626–534332 | 752 | 100 |
| *ARAF* | chrX:47422357–47430866 | 2134 | 100 |
| *BRAF* | chr7:140426284–140624513 | 2954 | 96.55 |
| *RAF1* | chr3:12626003–12660230 | 2347 | 98.13 |
| *MAP2K1* | chr15:66679676–66782963 | 1462 | 100 |
| *MAP2K2* | chr19:4090586–4123882 | 1423 | 99.79 |
| *MAPK1* | chr22:22123483–22221740 | 1243 | 100 |
| *MAPK3* | chr16:30127979–30134540 | 1375 | 100 |
| PI3 kinase complex | |  |  |
| *PIK3C3* | chr18:39535247–39661111 | 3436 | 97.24 |
| *PIK3R4* | chr3:130398149–130464072 | 4457 | 97.24 |
| *BECN1* | chr17:40962768–40975905 | 1888 | 92.85 |
| *ATG14* | chr14:55836327–55878550 | 1679 | 100 |
| Atg8 system |  |  |  |
| *MAP1LC3A* | chr20:33137772–33147712 | 518 | 100 |
| *ATG3* | chr3:112251536–112280385 | 1258 | 98.65 |
| *ATG4A* | chrX:107335047–107396952 | 1500 | 100 |
| *ATG4B* | chr2:242577120–242611689 | 1975 | 100 |
| *ATG4C* | chr1:63269448–63329840 | 1609 | 99.81 |
| *ATG4D* | chr19:10654757–10663753 | 1818 | 100 |
| *ATG7* | chr3:11340160–11605769 | 2734 | 100 |
| Atg12 system |  |  |  |
| *ATG5* | chr6:106494686–106764093 | 993 | 100 |
| *ATG10* | chr5:81283380–81549254 | 1053 | 100 |
| *ATG12* | chr5:115167491–115177400 | 780 | 100 |
| *ATG16L1* | chr2:234160464–234203006 | 2255 | 100 |
| *ATG16L2* | chr11:72525467–72540445 | 2617 | 100 |
| Regulation of the Atg1 kinase complex by TOR | |  |  |
| *ULK1* | chr12:132379537–132405916 | 3775 | 100 |
| *ATG13* | chr11:46665832–46693892 | 2026 | 99.21 |
| *RB1CC1* | chr8:53536221–53626703 | 5471 | 100 |
| *MTOR* | chr1:11167532–11319476 | 8790 | 100 |
| *RPTOR* | chr17:78519420–78938140 | 5148 | 99.44 |
| *DEPTOR* | chr8:120886077–121061953 | 1410 | 100 |
| *AKT1S1* | chr19:50373164–50379922 | 931 | 99.36 |
| *PTEN* | chr10:89623697–89725239 | 1912 | 90.74 |
| Atg9 complex |  |  |  |
| *ATG9A* | chr2:220085159–220092756 | 2802 | 100 |
| *ATG9B* | chr7:150712974–150721520 | 3116 | 100 |
| Mitophagy receptor | |  |  |
| *PINK1* | chr1:20960032–20977194 | 1906 | 100 |
| *PRKN* | chr6:161771121–163148710 | 2355 | 94.56 |
| *BNIP3* | chr10:133782018–133795515 | 904 | 100 |
| *BNIP3L* | chr8:26240637–26362845 | 813 | 100 |
| *FUNDC1* | chrX:44383434–44402088 | 568 | 100 |
| *OPTN* | chr10:13151113–13178876 | 2065 | 96.56 |
| *BCL2L13* | chr22:18121450–18210310 | 1903 | 99 |
| *CALCOCO2* | chr17:46919060–46940420 | 2048 | 97.12 |
| Colorectal cancer-related genes | |  |  |
| *APC* | chr5:112043405–112179833 | 9221 | 99.56 |
| *CTNNB1* | chr3:41265550–41280843 | 2665 | 100 |
| *ERBB2* | chr17:37855803–37884307 | 4545 | 96.99 |
| *SMAD4* | chr18:48573407–48604847 | 2034 | 99.95 |
| *PIK3CA* | chr3:178916604–178952162 | 3696 | 87.82 |
| *TP53* | chr17:7565247–7579922 | 1660 | 94.22 |

**Table S2.** Correlation between all SNVs and recurrence rate

|  | **Recurrence** | | **Non-recurrence** | |  |
| --- | --- | --- | --- | --- | --- |
| **Gene symbol** | **RefSeq (n)** | **AltSeq (n)** | **RefSeq (n)** | **AltSeq (n)** | ***p*-value** |
| RAS-MEK/ERK pathway | |  |  |  |  |
| *KRAS* | 22 | 5 | 42 | 15 | 0.5853 |
| *NRAS* | 26 | 1 | 55 | 2 | 1.0000 |
| *HRAS* | 27 | 0 | 57 | 0 | 1.0000 |
| *ARAF* | 24 | 3 | 54 | 3 | 0.3808 |
| *BRAF* | 22 | 5 | 46 | 11 | 1.0000 |
| *RAF1* | 25 | 2 | 54 | 3 | 0.6545 |
| *MAP2K1* | 27 | 0 | 57 | 0 | 1.0000 |
| *MAP2K2* | 24 | 3 | 51 | 6 | 1.0000 |
| *MAPK1* | 25 | 2 | 56 | 1 | 0.2407 |
| *MAPK3* | 25 | 2 | 55 | 2 | 0.5906 |
| PI3 kinase complex | |  |  |  |  |
| *PIK3C3* | 26 | 1 | 54 | 3 | 1.0000 |
| *PIK3R4* | 20 | 7 | 47 | 10 | 0.3946 |
| *BECN1* | 26 | 1 | 55 | 2 | 1.0000 |
| *ATG14* | 23 | 4 | 54 | 3 | 0.2041 |
| Atg8 system |  |  |  |  |  |
| *MAP1LC3A* | 27 | 0 | 57 | 0 | 1.0000 |
| *ATG3* | 1 | 26 | 8 | 49 | 0.2600 |
| *ATG4A* | 26 | 1 | 57 | 0 | 0.3214 |
| *ATG4B* | 0 | 27 | 1 | 56 | 1.0000 |
| *ATG4C* | 23 | 4 | 52 | 5 | 0.4596 |
| *ATG4D* | 26 | 1 | 54 | 3 | 1.0000 |
| *ATG7* | 24 | 3 | 54 | 3 | 0.3808 |
| Atg12 system | |  |  |  |  |
| *ATG5* | 19 | 8 | 51 | 6 | 0.0560 |
| *ATG10* | 8 | 19 | 17 | 40 | 1.0000 |
| *ATG12* | 27 | 0 | 54 | 3 | 0.5478 |
| *ATG16L1* | 16 | 11 | 28 | 29 | 0.4844 |
| *ATG16L2* | 20 | 7 | 41 | 16 | 1.0000 |
| Regulation of the Atg1 kinase complex by TOR | | | |  |  |
| *ULK1* | 0 | 27 | 0 | 57 | 1.0000 |
| *ATG13* | 27 | 0 | 52 | 5 | 0.1706 |
| *RB1CC1* | 4 | 23 | 2 | 55 | 0.0810 |
| *MTOR* | 21 | 6 | 40 | 17 | 0.6025 |
| *RPTOR* | 24 | 3 | 50 | 7 | 1.0000 |
| *DEPTOR* | 0 | 27 | 4 | 53 | 0.3002 |
| *AKT1S1* | 24 | 3 | 56 | 1 | 0.0955 |
| *PTEN* | 1 | 26 | 3 | 54 | 1.0000 |
| Atg9 complex | | |  |  |  |
| *ATG9A* | 23 | 4 | 47 | 10 | 1.0000 |
| *ATG9B* | 6 | 21 | 20 | 37 | 0.3142 |
| Mitophagy receptor | |  |  |  |  |
| *PINK1* | 18 | 9 | 21 | 36 | *0.0183 |
| *PRKN* | 10 | 17 | 24 | 33 | 0.8124 |
| *BNIP3* | 10 | 17 | 23 | 34 | 0.8151 |
| *BNIP3L* | 27 | 0 | 53 | 4 | 0.3002 |
| *FUNDC1* | 27 | 0 | 56 | 1 | 1.0000 |
| *OPTN* | 23 | 4 | 47 | 10 | 1.0000 |
| *BCL2L13* | 26 | 1 | 56 | 1 | 0.5422 |
| *CALCOCO2* | 3 | 24 | 3 | 54 | 0.3808 |
| Colorectal cancer-related genes | | | |  |  |
| *APC* | 0 | 27 | 0 | 57 | 1.0000 |
| *CTNNB1* | 24 | 3 | 56 | 1 | 0.0955 |
| *ERBB2* | 6 | 21 | 16 | 41 | 0.7909 |
| *SMAD4* | 22 | 5 | 48 | 9 | 0.7612 |
| *PIK3CA* | 23 | 4 | 39 | 18 | 0.1192 |
| *TP53* | 3 | 24 | 1 | 56 | 0.0955 |

**Table S3.** Correlation between ClinVar-based pathogenic SNVs and recurrence rate

|  | **Recurrence** | | **Non-recurrence** | |  |
| --- | --- | --- | --- | --- | --- |
| **Gene symbol** | **RefSeq (n)** | **AltSeq (n)** | **RefSeq (n)** | **AltSeq (n)** | ***p*-value** |
| *APC* | 8 | 19 | 19 | 38 | 0.8063 |
| *BRAF* | 26 | 1 | 53 | 4 | 1.0000 |
| *CTNNB1* | 27 | 0 | 57 | 0 | 1.0000 |
| *KRAS* | 22 | 5 | 45 | 12 | 1.0000 |
| *NRAS* | 27 | 0 | 55 | 2 | 1.0000 |
| *PIK3CA* | 25 | 2 | 45 | 12 | 0.2082 |
| *PTEN* | 27 | 0 | 56 | 1 | 1.0000 |
| *RAF1* | 27 | 0 | 56 | 1 | 1.0000 |
| *SMAD4* | 25 | 2 | 54 | 3 | 0.6545 |
| *TP53* | 10 | 17 | 30 | 27 | 0.2431 |
